# Conditions for escape of a rotor in a rotary nanobearing from short triple-wall nanotubes

**DOI:** 10.1038/s41598-017-07184-x

**Published:** 2017-07-28

**Authors:** Jiao Shi, Ling-Nan Liu, Kun Cai, Qing-Hua Qin

**Affiliations:** 1 College of Water Resources and Architectural Engineering, Northwest A&F University, Yangling, 712100 China; 2 Research School of Engineering, the Australian National University, Canberra, ACT 2601 Australia

## Abstract

In a short nanobearing system made from carbon nanotubes, the rotor with high rotational frequency may escape from the stator, which may cause a stability problem to the system of a nanodevice with such a nanobearing. In the present work, nanobearings with tri-walled nanotubes are investigated to reveal the conditions for the moving away of the free inner tube from the high-speed rotating middle tube. Experimental results show that the escape happens when the radii difference between the two rotors is larger than 0.34 nm and the rotational frequency of the middle tube is higher than a critical value. And before the escape occurs, the rotational frequency of the inner tube is lower than this critical value. Due to the radii difference being larger than 0.34 nm, the two rotors are non-coaxial, and the centrifugal force of the inner tube results in strong radial and axial interactions between the edges of the two rotors. When the relative sliding speed is relatively high, an edge of the inner rotor will pass through the potential barrier at the adjacent edge of the middle rotor, and further escape from the middle rotor occurs. The selection of a longer middle rotor with smaller radius can increase the critical rotational frequency of the middle rotor.

## Introduction

Carbon nanotubes (CNTs)^1, 2^ are popular in the design of such nanodevices as nanomotors^3–13^, nanobearings^14–16^, nanoswitch^17, 18^, nano strain sensors^19–21^, and nanooscillators^22–27^, due to their distinguished mechanical properties. For example, the in-plane *sp* ^2^-*sp* ^2^ covalent bonds lead to ultrahigh modulus and strength of shell^28–31^, which benefits the stability of the nanodevices having CNTs as components under ultrahigh loading. On the other hand, the delocalized π-electrons result in ultralow friction between shells^14, 16, 25, 32^. The ultralow friction implies that the relative motion between neighbor tubes is easy to be actuated. Besides, the kinetic energy dissipation^27^ will be very small or can be easily made up by external fields^4, 6, 7, 15, 33–35^. Commonly, there exist two types of relative motion, i.e., translation and rotation. Translation is the relative sliding along tube axes of multi-walled CNTs. If the moving CNTs carry cargoes during sliding along tube axis, the nano mechanism can be used as a linear nanomotor^4, 6, 9^. For instance, Cumings and Zettl^14^ found that the inner tubes in multi-walled CNTs can be shrunk back into the outer tubes when they are pulled out partly and further released. Barreiro *et al*.^4^ fabricated a nanodevice in which a cargo was attached to an ablated outer tube of multi-walled carbon. When the temperature sloped up or down along the axis inner tubes, the cargo generated two types of motion: translation and rotation. For the moving CNTs having very high periodically reciprocating axial sliding, the nanodevice is called a nano-oscillator. Inspired by Cumings and Zettl’s experiments^14^, Zheng and Jiang^25^ built a naon-oscillator model from multi-walled CNTs. Further, Legoas *et al*.^26^ studied the feasibility of a giga-Hertz oscillator using molecular dynamics simulations. Guo *et al*.^27^ estimated the energy dissipation of the oscillator from either commensurate or incommensurate double-walled CNTs. Cai *et al*.^22^ found that the axial oscillation or the inner tube can be excited when releasing the high speeding rotating rotor in the stator. Comparing to the axially sliding, the circumferential sliding between neighbor tubes is not easy to be excited. When a nanodevice has such motion which can be kept for a period of long time, we call the nanodevice a rotary nanomotor. Fennimore *et al*.^3^ constructed a nanoscale actuator, in which the multi-walled CNTs were used as shaft of the rotating part. Similarly, Bourlon *et al*.^15^ fabricated a rotary actuator which was able to be driven by an external electric field. Tu and Hu^11^ simulated a rotary nanomotor from CNTs which was driven by axially varying voltage. Kang and Hwang^12^ suggested to driving the rotation of a CNT-based rotor by nano-fluids. In 2008, Wang *et al*.^5^ developed a rotary nanomotor based on electron tunneling effect. The rotor was able to be actuated to rotate by an external electric field. In 2014, Cai *et al*.^7^ developed a rotary nanomotor from double-walled nanotubes. In the nanomotor, the rotor (inner tube) was able to be excited to rotate within the fixed outer tube at a canonical NVT ensemble. Recently, a thermally-driven rotary nanomotor from CNTs was reported which can be controlled well with specified rotational direction and speed^10^. And they also proposed possible approaches to observe^36^ or even measure the rotation^37, 38^.

Benefitted from the success of rotary nanomotor, the rotation transmission system (RTS) was presented in 2015^39, 40^. A RTS contains two major components, a rotary nanomotor and a nanobearing. The rotor in the nanobearing can be driven to rotate by the rotary nanomotor when their distance is less than 1 nm. Due to different factors such as temperature^41^ and geometry of tubes^42^ in nanobearing, the rotational frequency of the rotor may be different obviously from that of the nanomotor. Hence, such nano device can be used to shift the output rotation of the nanomotor. Results showed that the rotor can obtain a higher frequency than the nanomotor does on certain conditions^43^. And not only the rotational frequency of motor can be changed by the nanobearing, but also the direction of rotation can be transformed^44^.

When a nanobearing is adopted to transform the rotation of nanomotor, the stability of the naonbearing should be maintained during the operation. In our recent study, we found that the rotor may escape from the motor or stator when the input rotational frequency is high enough and the rotor is far longer than the motor-tube^45, 46^. Results demonstrated that the eccentric rotation of rotor is the major reason for its escape from stator. One may have the questions: will a short rotor escape from the stator? how to avoid the escape? To answer the questions, we consider a nanobearing from tri-walled CNTs, and study the dynamics stability of the nanosystem.

## Models and Methodology

### Models

#### Methodology

**Figure 1:**
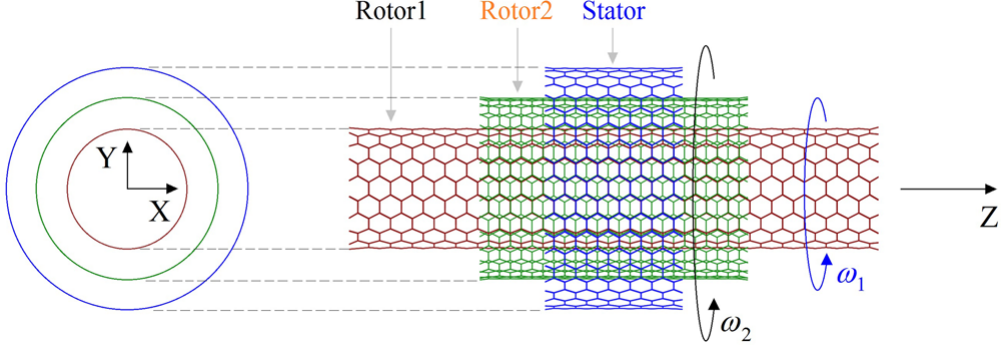
Schematic model of a rotary nanobearing from TWNTs with given middle and outer tubes. The inner tube (Rotor1) with a length of L1 nearby 6 nm in each model will act as rotor, which will be driven to rotate by the rotating middle tube (Rotor2) with a length of L2 = ~2.828 nm. The outer tube with a length of L3 = ~1.353 nm will be fixed as a stator in each model. All tube ends are hydrogenated. The input rotational frequency of Rotor2 is *ω*_2_, which leads to the output rotational frequency of Rotor1, i.e., *ω*_1_. In the present experiments, two types of nanobearings are involved, and the details are listed in Table 1.

In the present numerical experiments, molecular dynamics simulation approach is adopted to find the dynamic response of the inner tube in the nanobearing shown in Fig. 1. Each simulation contains following steps:

Step 1. Build the nanobearing system with specified CNTs hydrogenated on their edges;

Step 2. Reshape the nanosystem by minimizing the potential energy using steepest decent method;

Step 3. Relax the nanosystem at a canonical (NVT) ensemble with T = 300 K for 200 ps. In relaxation, the atoms at edges of both rotors are fixed, and the whole stator is fixed;

Step 4. Release both rotors after relaxation, and set the right edge of Rotor2 rotating along the tube axis of the stator for a constant period;

Step 5. Record the dynamic response of Rotor1 for postprocessing.

The simulations are carried out in the open source code LAMMPS^47^. In the simulation, interaction among neighbor carbon and/or hydrogen atoms is estimated by AIREBO potential^48^. The time step is of 0.001 ps. The maximal number of steps is 10,000,000.

On the other hand, the bi-section method will be used to find the critical rotational frequency of *ω*_2_. Here we define L(*ω*_2_) as the axial distance between the mass centers of the left edges of both rotors when Rotor2 has rotational frequency of *ω*_2_. The flowchart of the method is mathematically written as follows.

- Find the initial interval of *ω*_2_, e.g., [a, b], at 10 ns, L(a) < L1−L2, i.e., Rotor1 rotates stably. At anytime, when L(b) > L1, Rotor1 escapes from Rotor2;
- Let c = (a + b)/2, calculate L(c);
- If L(c) < L1−L2 at 10 ns or *ω*_1_ = *ω*_2_ for 2 ns, let a = c; or L(c) > L1, let b = c;
- if (b-a) < 2 GHz, go to e); otherwise, go to b);
- Stop and write down *ω*_2_^cr^ = c and the related data.

In the calculation, conditions L(c) < L1−L2 and L(c) > L1 are examined at every 200,000 steps.

## Numerical Results and Discussion

### The output rotation or Rotor1 in the A-type nanobearing

**Figure 2:**
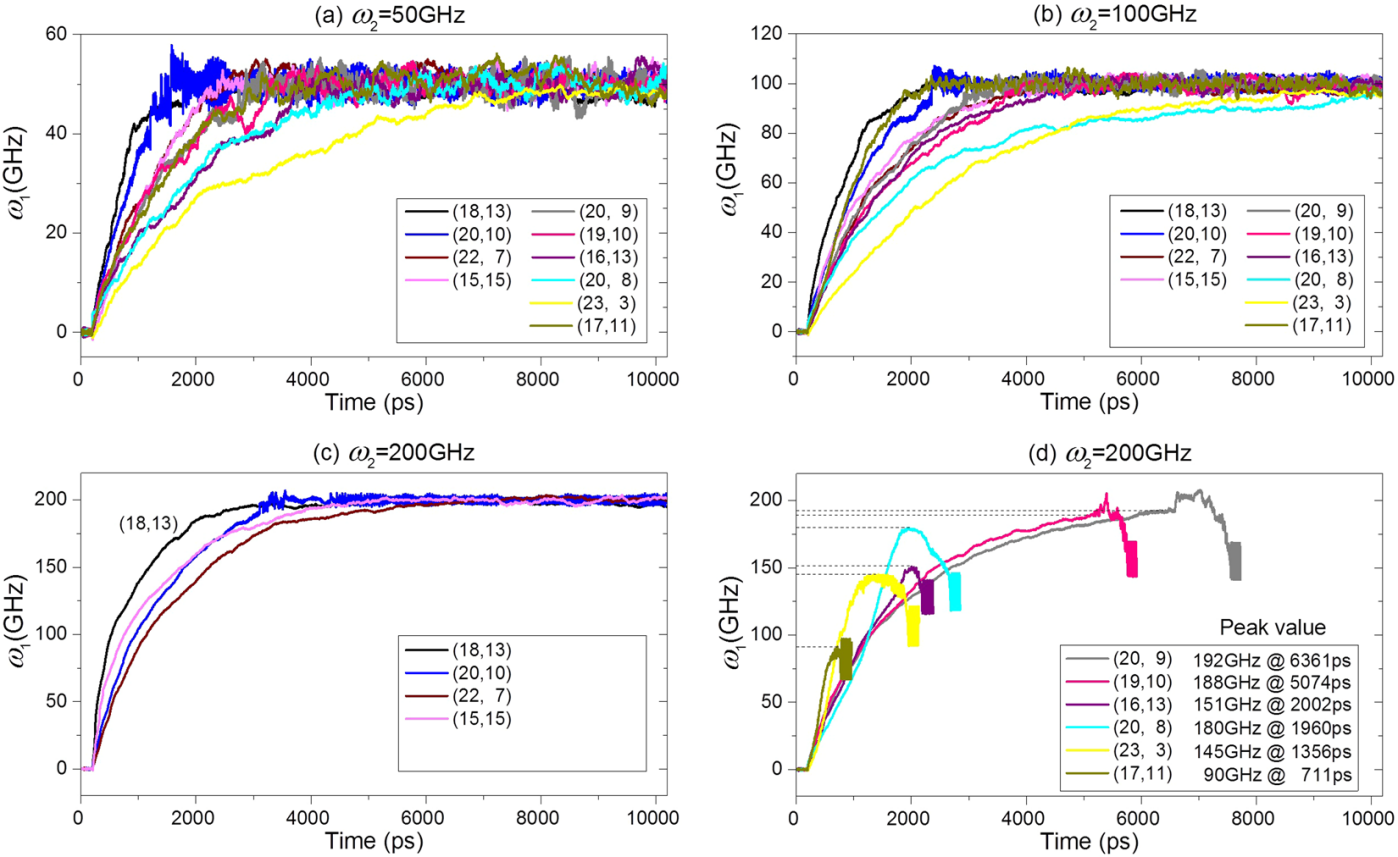
The history curves of *ω*_1_, the output rotational frequency of Rotor1 when driven by Rotor2 with different input rotational frequency of *ω*_2_ in the A-type model.

When specifying a constant rotational frequency onto the middle tube (Rotor2) in the nanobearing shown in Fig. 1, the inner tube (Rotor1) will be driven to rotate quickly. For instance, in the case of the bearing from (n_1_, m_1_)/(20, 20)/(25, 25), the rotational histories of Rotor1 are shown in Fig. 2. When the input rotational frequency of Rotor2 is 50 GHz (as shown in Fig. 2a), the final rotational frequency of Rotor1 with any radius will tend to be identical to 50 GHz after a period of rotational acceleration. Clearly, the Rotor1 with higher radius, e.g., (18, 13) needs shorter time on the acceleration. The reason is that the intertube friction is higher when the radii difference is smaller. The curve of rotational frequency of the Rotor1 from (17, 11) CNT looks peculiar, which indicates that the tube needs shorter time to rotate synchronously with Rotor2. If the input rotational frequency of Rotor2 increases to 100 GHz, the duration of the acceleration process of Rotor1 shows monotonously decreasing with respect to its radius. Further, the curve related to (17, 11) also implies that the Rotor1 does not obey the rule.

When *ω*_2_ = 200 GHz, the curves of rotational frequency in Fig. 2c and d are obviously different. From the curves shown in Fig. 2c, we know that the Rotor1 with larger radius, i.e., the radii difference between both rotors is less than 0.335 nm of the equilibrium distance between two CNTs, will rotate almost synchronously with Rotor2. However, when the radius of Rotor1 becomes smaller, e.g., (17, 11)’s radius is ~0.4 nm smaller than that of Rotor2, the peak value of *ω*_1_ is less than 200 GHz for Rotor2. Besides, the value of *ω*_1_ drops quickly soon after arriving at the peak value. Later, the value of *ω*_1_ shows periodic fluctuation. When checking the configuration of the nanobearing, we find that Rotor1 has escaped from Rotor2 (Movie 1).

From the discussion above, a conclusion can be made that when *ω*_2_ is between 50–200 GHz, the radii difference between the two rotors being less than 0.34 nm will result in a stable synchronous rotation of Rotor1 in the rotating Rotor2. If the radii difference is larger than 0.34 nm, Rotor1 will escape from Rotor2. In general, the Rotor1 with smaller radius requires shorter time to escape.

**Figure 3:**
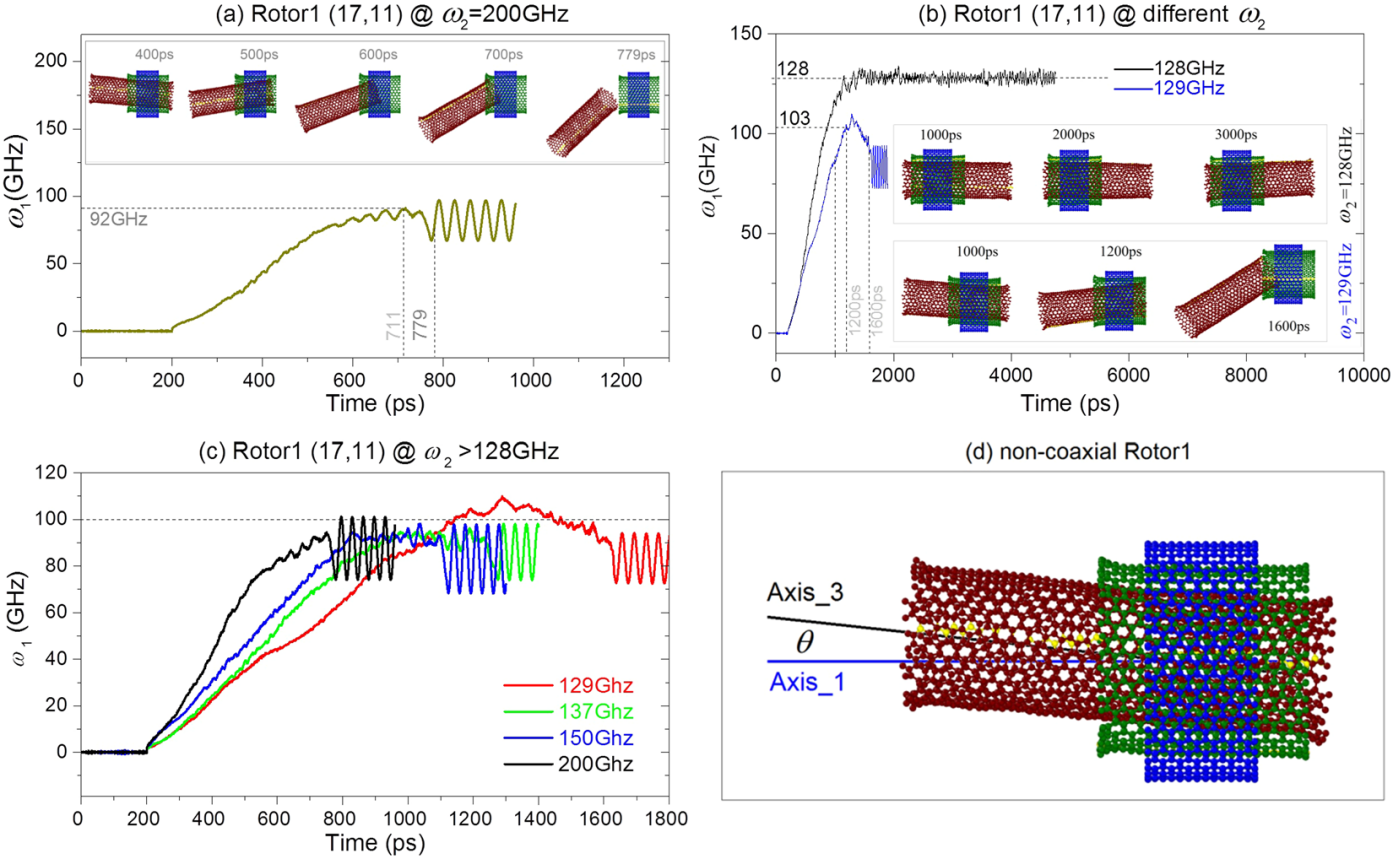
Curves of *ω*_1_ and the snapshots of the nanobearing from TWNT (17, 11)/(20, 20)/(25, 25) when driven by Rotor2. (**a**) *ω*_2_ = 200 GHz. (**b**) *ω*_2_ is nearby the critical value, i.e., 128 GHz. (**c**) *ω*_2_ > 128 GHz (the values appear in bi-section process). (**d**) Rotor1 shows non-coaxial with Stator.

**Figure 4:**
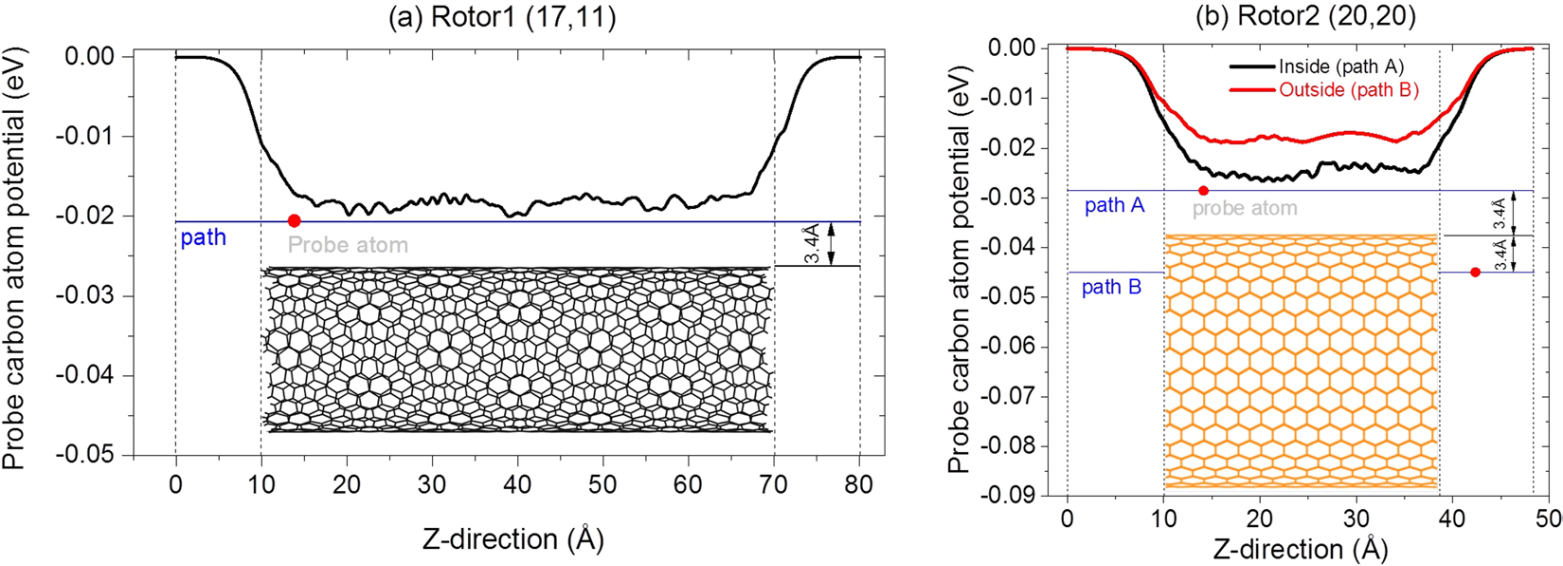
Potential of a probe of carbon atom (red point) when moving along the path (blue line) which is 3.4 Å away from a single tube. (**a**) Tube is Rotor1 (17,11), (**b**) tube is Rotor2 (20, 20). The tubes are chosen from the system after 200 ps of relaxation. Inserts are standard tubes before relaxation.

The discovery of the escape of Rotor1 from Rotor2 incurs the exploring of the escape mechanism. Take Rotor1 from (17, 11) CNT for example, it is still in stable state at 400 ps when driven by the Rotor2 with 200-GHz (the insert snapshot in Fig. 3a). Further rotational acceleration of Rotor1 leads to eccentric rotation^45, 46^ and the right edge of the tube has overcome the potential barrier (Fig. 4)^49^ at the right edge of Rotor2 at 500 ps. At 700 ps, the right edge of Rotor1 has arrived at the left end of Rotor2. Meanwhile, the value of *ω*_1_ is very close to the peak value. Finally, Rotor1 escapes from the left edge of Rotor2 at 779 ps. After escaping from Rotor2, Rotor1 is under free rigid-body motion state, i.e., there is neither external force nor external moment being applied on Rotor1 (Movie 1). Hence, the projection of the rotational frequency of Rotor1 along Z-axis (tube axis of Stator) varies periodically.

Considering the curves shown in Fig. 2, we predict that there must have a critical value of *ω*_2_ between 100–200 GHz, the Rotor1 with smaller radius will be in stable synchronous rotational state if *ω*_2_ is not larger than the critical value, or Rotor1 will escape from Rotor2 when *ω*_2_ is larger than the critical value. Using bi-section approach, we can find the critical value of *ω*_2_. For example, using bi-section approach, we find that the critical value of *ω*_2_ is ~128 GHz with respect to the Rotor from (17, 11) CNT in the current model. When *ω*_2_ = 128 GHz, Rotor1 is in stable rotational state (see the insert snapshot in Fig. 3b). However, Rotor1 escapes from Rotor2 after 1600ps when *ω*_2_ = 129 GHz. Comparing the curves with respect to (17, 11) CNT in Fig. 2d and Fig. 3b, we find that the peak value of *ω*_1_ is ~103 GHz at 1200 ps, which is smaller than the critical value of *ω*_2_, i.e., 128 GHz. Besides, the peak value of *ω*_1_ is nearby 100 GHz for any *ω*_2_ > 128 GHz (Fig. 3c). Hence, the peak value of *ω*_1_ depends mainly on the geometry of the system and slightly on *ω*_2_. Meanwhile, the larger value of *ω*_2_ leads to escape of Rotor1 happening earlier.

**Figure 5:**
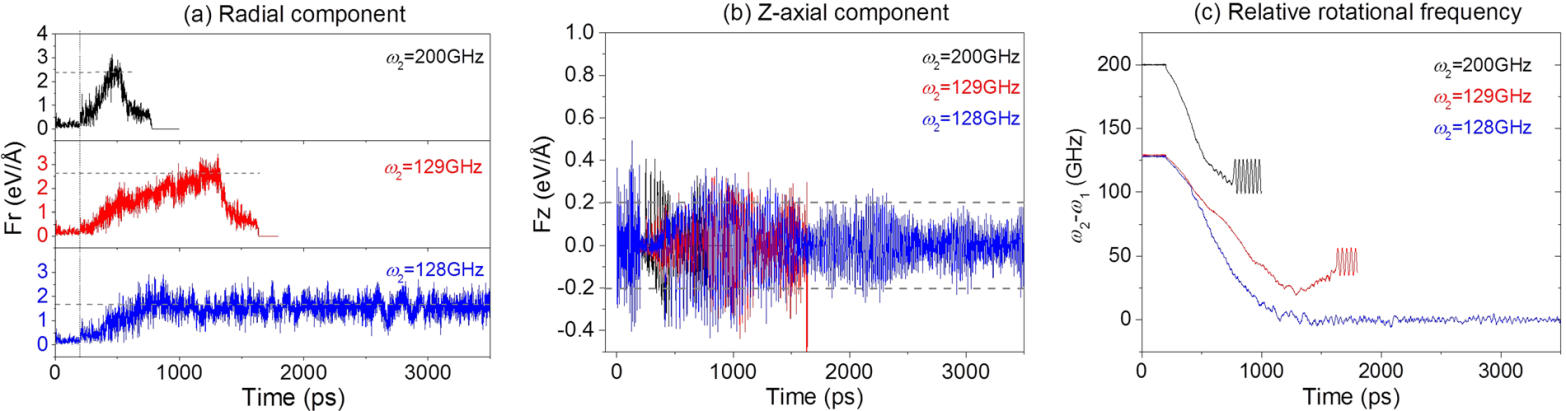
Comparisons of the radial centrifugal force, axial force of Rotor1 (17, 11), and relative rotational frequency between both rotors when driven by Rotor2 with different values of *ω*_2_.

Comparing the snapshots at different *ω*_2_ nearby the critical value (Fig. 3b), we find that the left edge of Rotor1 is kept outside of the left edge of Rotor2 with *ω*_2_ = 128 GHz. Even if the rotational frequency of Rotor1 is identical to that of Rotor2, the left edge of Rotor1 does not enter into Rotor2. However, when *ω*_2_ is 129 GHz, the right edge of Rotor1 can easily go into Rotor2 even if the rotational frequency of Rotor1 is smaller than 103 GHz. What is the reason for this phenomenon? Before answering the question, we draw the radial and z-axial components of centrifugal force at the mass center of Rotor1 which is driven by Rotor2 with different *ω*_2_ (Fig. 5a,b). One can find that the value of radial component (Fr) is smaller than 2 eV/Å when *ω*_2_ = 128 GHz. If *ω*_2_ is larger than 128 GHz, e.g., 129 GHz, the value of Fr is larger than 2 eV/Å. But it takes longer time to become larger than 2 eV/Å as comparing to the case of *ω*_2_ = 200 GHz. Larger radial force leads to larger angle between tube axes of both rotors (e.g., see the snapshot at 400 ps when *ω*_2_ = 200 GHz in Fig. 3a,d). Meanwhile, the contact force between both rotors at their right edges becomes larger. It is also necessary to demonstrate that the relative sliding between both rotors are larger either (Fig. 5c). Hence, the right edge of Rotor1 can pass through the edge barrier of Rotor2 (Fig. 4) if the axial force (Fz) exists (Fig. 5b). As the right edge of Rotor1 enters into the Rotor2, the relative sliding between both rotors becomes easier. Within half of nanosecond, the right edge of Rotor1 can run away from the left end of Rotor2. Now, Rotor1 escapes completely from Rotor2. This is the major reason for the escape of Rotor1 from Rotor2. It also demonstrates that eccentric rotation of the rotor is the incentive of escape. If there is no eccentric rotation of Rotor1, it cannot escape from Rotor2. For a short nanobearing from CNTs, it is difficult to control the eccentric rotation. But for a long nanobearing, it is feasible to avoid eccentric rotation^45^.

### The output rotation of Rotor1 in the B-type motor

When the radii difference between the two rotors is larger than 0.34 nm, the angle between tube axes depends on the two factors: the radii and the length of Rotor2 (L2 < L1). When the length of Rotor2 is fixed, the angle becomes smaller at the same radii difference (>0.34 nm) for smaller radius of Rotor2. In the discussion above, the CNT (20, 20) is used for Rotor2. Now if we choose (15, 15) tube with the same length of ~2.828 nm as a new Rotor2 in the system, and respectively, (20, 20) as Stator, can we find the critical value of *ω*_2_, over which the inner tube (Rotor1) will escape from Rotor2? Clearly, with the same radius difference, the angle between tube axes becomes smaller. In this section, this question will be answered using the experiments considering the B-type model in Table 1.

**Table 1:** Parameters of the inner tube in the two types of nanobearing models from TWNTs.

A-type model: (n_1_, m_1_)/(20, 20)/(25, 25)					B-type model: (n_1_, m_1_)/(15, 15)/(20, 20)				
(n_1_, m_1_)	Radius	∆r	L1	Atoms	(n_1_, m_1_)	Radius	∆r	L1	Atoms
(18, 13)	1.055	0.300	5.978	1514C + 62H	16, 4	0.717	0.300	5.810	1000C + 40H
(20, 10)	1.035	0.320	5.904	1480C + 60H	14, 6	0.696	0.321	5.927	988C + 40H
(22, 7)	1.026	0.330	5.989	1466C + 58H	13, 7	0.688	0.329	5.913	976C + 40H
(15, 15)	1.017	0.339	5.780	1440C + 60H	10, 10	0.678	0.339	5.780	960C + 40H
(20, 9)	1.007	0.350	5.954	1432C + 58H	14, 5	0.668	0.349	5.944	950C + 38H
(19, 10)	0.999	0.357	5.869	1402C + 58H	13, 6	0.659	0.359	5.930	936C + 38H
(16, 13)	0.985	0.371	5.977	1412C + 58H	16, 1	0.647	0.370	5.982	920C + 34H
(20, 8)	0.978	0.378	6.037	1416C + 56H	14, 4	0.641	0.376	5.924	908C + 36H
(23, 3)	0.965	0.392	6.040	1386C + 52H	16, 0	0.627	0.390	5.822	896C + 32H
(17, 11)	0.957	0.400	6.015	1378C + 56H	14, 3	0.615	0.402	5.905	866C + 34H

The radius of (20, 20) middle tube is ~1.355 nm. ∆r is the radii difference between the inner and middle tubes. Dimension unit: nm.

**Figure 6:**
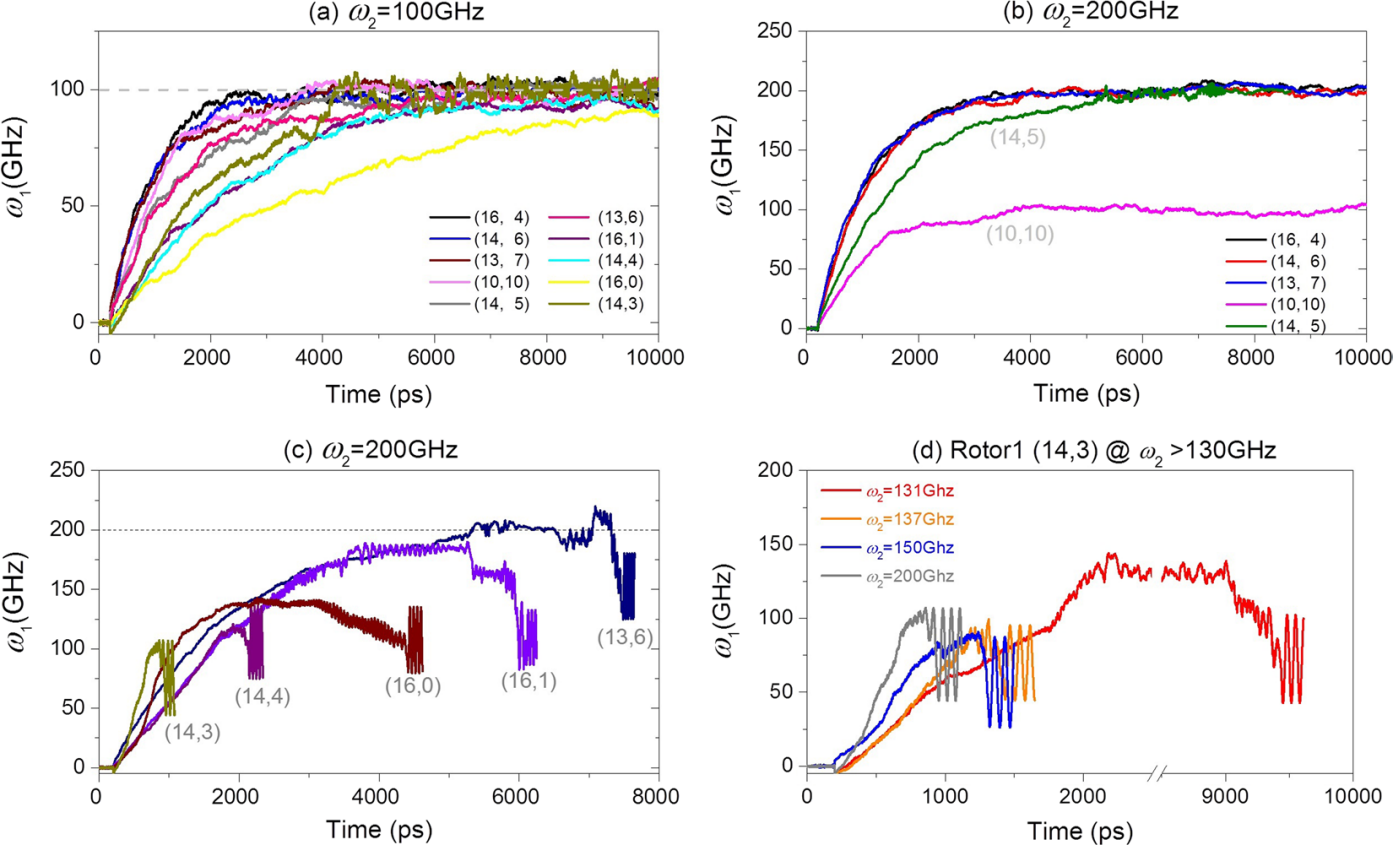
In the B-type model, the history curves of *ω*_1_ of Rotor1 when driven by Rotor2 with different input rotational frequency of *ω*_2_.

From Fig. 2d, Rotor1 with smaller radius needs shorter time to escape from Rotor2. Hence, we predict that Rotor1 with the same radius difference (>0.34 nm) needs longer time to escape from Rotor2. In Fig. 6, the history curves of the rotational frequency of Rotor1 are given with respect to different *ω*_2_. In Fig. 6a, the value of *ω*_1_ tends to be identical to *ω*_2_, i.e., 100 GHz. It implies that no Rotor1 escapes from Rotor2. In Fig. 6b, for the five cases of (16, 4), (14, 6), (13, 7), (10, 10) and (14, 5), Rotor1 rotates in stable state after about 5 ns. Two phenomena need to be demonstrated. One is that Rotor1 from (10,10) tube rotates at ~100 GHz. The other is that Rotor1 from (14, 5) with radius difference of ~0.349 nm >0.34 nm (Table 1) does not escape from (15, 15) Rotor2, as well. Comparing to Rotor1 from (20, 9) escaping from (20,20) Rotor2, the ratio between radii difference and length of Rotor2 is important to the stability of the rotational nanosystem. In Fig. 6c, the above prediction of escaping time of Rotor1 is verified. But among the results, the (16, 0) Rotor 1, whose radius is shorter than that of the (14, 4) Rotor 1, needs longer time to escape from Rotor2 (Movie 2). It implies that zigzag nanotube escaping from Rotor2 is not easier than the chiral nanotubes with the same radius due to the regular edge. In Fig. 6d, we provide four curves of *ω*_1_ of Rotor1 driven by Rotor2 whose rotational frequency is larger than the critical value of 130 GHz. When *ω*_2_ is larger than 137 GHz, Rotor1 needs no more than 2000ps to escape from Rotor2. The curve of *ω*_2_ = 131 GHz implies that Rotor1 may escape even after a long period of time of stable rotation.

## Conclusions

For exploring the conditions of Rotor1escaping from Rotor2, two groups of samples are considered for the simulation. According to the numerical analysis above, some interesting conclusions are made which is helpful for designing short tri-walled rotary nanobearing.

1. For a short nanobearing, a long inner tube can rotate in stable state in the middle tube when the radius difference between them is smaller than 0.34 nm. In this case the inner tube may escape from the Rotor2 with larger rotational frequency (*ω*_2_);
2. There is a critical value of rotational frequency of Rotor2 at which Rotor1 can escape from Rotor2. In other words, escape happens once *ω*_2_ is larger than the critical value. The peak value of *ω*_1_ is obviously smaller than *ω*_2_;
3. Generally, the higher radius difference between both rotors leads to shorter time for Rotor1 to escape from Rotor2;
4. For the same radius difference (>0.34 nm) between the two rotors, Rotor1 needs longer time to escape from the Rotor2 with the same length but smaller radius.

From the mechanism of escape of the inner tube from the middle tube, one approach to prevent the escape is to enhance the edge potential barriers of the two tubes if the geometry and chirality of the tubes in the nanobearing have been specified.

## Electronic supplementary material

video 1

video 2

Supplementary Information

## References

[1] Iijima S (1991). Helical microtubules of graphitic carbon. Nature.

[2] Zou J, Ji B, Feng X-Q, Gao H (2006). Self-assembly of single-walled carbon nanotubes into multiwalled carbon nanotubes in water: molecular dynamics simulations. Nano Lett..

[3] Fennimore A (2003). Rotational actuators based on carbon nanotubes. Nature.

[4] Barreiro A (2008). Subnanometer motion of cargoes driven by thermal gradients along carbon nanotubes. Science.

[5] Wang B, Vuković L, Král P (2008). Nanoscale rotary motors driven by electron tunneling. Phys. Rev. Lett..

[6] Zambrano HA, Walther JH, Jaffe RL (2009). Thermally driven molecular linear motors: A molecular dynamics study. J. Chem. Phys..

[7] Cai K, Li Y, Qin QH, Yin H (2014). Gradientless temperature-driven rotating motor from a double-walled carbon nanotube. Nanotechnology.

[8] Cai K, Yu J, Yin H, Qin QH (2015). Sudden stoppage of rotor in a thermally driven rotary motor made from double-walled carbon nanotubes. Nanotechnology.

[9] Chen J (2015). Impeded mass transportation due to defects in thermally driven nanotube nanomotor. J. Phys. Chem. C.

[10] Cai K, Wan J, Qin QH, Shi J (2016). Quantitative control of a rotary carbon nanotube motor under temperature stimulus. Nanotechnology.

[11] Tu Z, Hu X (2005). Molecular motor constructed from a double-walled carbon nanotube driven by axially varying voltage. Phys. Rev. B.

[12] Kang JW, Hwang HJ (2004). Nanoscale carbon nanotube motor schematics and simulations for micro-electro-mechanical machines. Nanotechnology.

[13] Hamdi M, Subramanian A, Dong L, Ferreira A, Nelson BJ (2013). Simulation of rotary motion generated by head-to-head carbon nanotube shuttles. Mechatronics, IEEE/ASME Transactions on.

[14] Cumings J, Zettl A (2000). Low-friction nanoscale linear bearing realized from multiwall carbon nanotubes. Science.

[15] Bourlon B, Glattli DC, Miko C, Forró L, Bachtold A (2004). Carbon nanotube based bearing for rotational motions. Nano Lett..

[16] Cook EH, Buehler MJ, Spakovszky ZS (2013). Mechanism of friction in rotating carbon nanotube bearings. J. Mech. Phys. Solids.

[17] Deshpande VV (2006). Carbon nanotube linear bearing nanoswitches. Nano Lett..

[18] Subramanian, A., Dong, L. X., Nelson, B. J. & Ferreira, A. Supermolecular switches based on multiwalled carbon nanotubes. *Appl. Phys. Lett*. **96** (2010).

[19] Qiu W, Kang YL, Lei ZK, Qin QH, Li Q (2009). A new theoretical model of a carbon nanotube strain sensor. Chinese Phys. Lett..

[20] Qiu W, Kang YL, Lei ZK, Qin QH, Li Q (2010). Experimental study of the Raman strain rosette based on the carbon nanotube strain sensor. J. Raman Spectrosc..

[21] Qiu W, Li Q, Lei ZK, Qin QH, Deng WL (2013). The use of a carbon nanotube sensor for measuring strain by micro-Raman spectroscopy. Carbon.

[22] Cai K, Yin H, Qin QH, Li Y (2014). Self-excited oscillation of rotating double-walled carbon nanotubes. Nano Lett..

[23] Kang JW, Kim K-S, Hwang HJ, Kwon OK (2010). Molecular dynamics study of effects of intertube gap on frequency-controlled carbon-nanotube oscillators. Phys. Lett. A.

[24] Ansari R, Sadeghi F, Ajori S (2013). Continuum and molecular dynamics study of C 60 fullerene–carbon nanotube oscillators. Mech. Res. Commun..

[25] Zheng Q, Jiang Q (2002). Multiwalled carbon nanotubes as gigahertz oscillators. Phys. Rev. Lett..

[26] Legoas S (2003). Molecular-dynamics simulations of carbon nanotubes as gigahertz oscillators. Phys. Rev. Lett..

[27] Guo W, Guo Y, Gao H, Zheng Q, Zhong W (2003). Energy dissipation in gigahertz oscillators from multiwalled carbon nanotubes. Phys. Rev. Lett..

[28] Qian D, Wagner GJ, Liu WK, Yu M-F, Ruoff RS (2002). Mechanics of carbon nanotubes. Appl. Mech. Rev..

[29] Bichoutskaia E, Heggie MI, Popov AM, Lozovik YE (2006). Interwall interaction and elastic properties of carbon nanotubes. Phys. Rev. B.

[30] Zhang H, Cai K, Wang L (2008). Deformation of single-walled carbon nanotubes under large axial strains. Mat. Lett..

[31] Qin Z, Qin QH, Feng XQ (2008). Mechanical property of carbon nanotubes with intramolecular junctions: molecular dynamics simulations. Phys. Lett. A.

[32] Zhang R (2013). Superlubricity in centimetres-long double-walled carbon nanotubes under ambient conditions. Nat. Nanotechnol..

[33] Legoas SB (2004). Gigahertz nanomechanical oscillators based on carbon nanotubes. Nanotechnology.

[34] Rivera JL, McCabe C, Cummings PT (2003). Oscillatory behavior of double-walled nanotubes under extension: a simple nanoscale damped spring. Nano Lett..

[35] Ershova OV (2010). Nanotube-based nanoelectromechanical systems: Control versus thermodynamic fluctuations. Phys. Rev. B.

[36] Cai K, Yu J, Wan J, Yin H, Qin QH (2016). Configuration jumps of rotor in a nanomotor from carbon nanostructures. Carbon.

[37] Cai K, Yu J, Shi J, Qin QH (2016). A method for measuring rotation of a thermal carbon nanomotor using centrifugal effect. Sci. Rep..

[38] Cai K, Yu J, Liu L, Shi J, Qin QH (2016). Rotation measurements of a thermally driven rotary nanomotor with a spring wing. Phys. Chem. Chem. Phys..

[39] Cai K, Yin H, Wei N, Chen Z, Shi J (2015). A stable high-speed rotational transmission system based on nanotubes. Appl. Phys. Lett..

[40] Yin H, Cai K, Wei N, Qin QH, Shi J (2015). Study on the dynamics responses of a transmission system made from carbon nanotubes. J. Appl. Phys..

[41] Cai K, Zhang X, Shi J, Qin QH (2015). Temperature effects on a motion transmission device made from carbon nanotubes: a molecular dynamics study. RSC Adv..

[42] Yin H, Cai K, Wan J, Gao Z, Chen Z (2016). Dynamic response of a carbon nanotube-based rotary nano device with different carbon-hydrogen bonding layout. Appl. Surf. Sci..

[43] Cai K, Cai H, Ren L, Shi J, Qin QH (2016). Over-Speeding Rotational Transmission of a Carbon Nanotube-Based Bearing. J. Phys. Chem. C.

[44] Gao ZL (2017). Effect of hydrogenation and curvature of rotor on the rotation transmission of a curved nanobearing. Comp. Mater. Sci..

[45] Cai K, Yu JZ, Shi J, Qin QH (2017). Robust rotation of rotor in a thermal driven nanomotor. Sci. Rep..

[46] Cai K, Zhang X, Shi J, Qin QH (2017). Rotation-excited perfect oscillation of a tri-walled nanotube-based oscillator at ultralow temperature. Nanotechnology.

[47] Plimpton S (1995). Fast parallel algorithms for short-range molecular dynamics. J. Comp. Phys..

[48] Stuart SJ, Tutein AB, Harrison JA (2000). A reactive potential for hydrocarbons with intermolecular interactions. J. Chem. Phys..

[49] Guo Z, Chang T, Guo X, Gao H (2011). Thermal-induced edge barriers and forces in interlayer interaction of concentric carbon nanotubes. Phys. Rev. Lett..

