# Supplementary material for: Conditions for escape of a rotor in a rotary nanobearing from short triple-wall nanotubes
Source: Sci Rep. 2017 Jul 28;7:6772. doi: 10.1038/s41598-017-07184-x (PMC5533743; doi:10.1038/s41598-017-07184-x)
Supplement: Supplementary file 3 — Supplementary Information [file 41598_2017_7184_MOESM3_ESM.pdf]

Conditions for escape of a rotor in a rotary nanobearing from short triple-wall nanotubes

Jiao Shi [1], Ling-Nan Liu [1], Kun Cai [1,2*], Qing-Hua Qin [21]

[1] *College of Water Resources and Architectural Engineering, Northwest A&F University, Yangling 712100, China*

[2] *Research School of Engineering, the Australian National University, ACT, 2601, Australia*

Supplementary information

Movies:

Movie1-(17,11)-200GHz-during [400, 800]ps.avi;

Movie2-(16,0)-200GHz-during [4200, 4600]ps.avi;

[1] Correspondence to: kuncai99@163.com (K. Cai); qinghua.qin@anu.edu.au (Q.H. Qin)
